# Direct Characterization of Free Solutal Convection in Porous Rocks for CO_2_ Storage Applications

**DOI:** 10.1021/acs.est.4c10183

**Published:** 2025-03-03

**Authors:** Anna-Maria Eckel, Andrea Rovelli, Ronny Pini

**Affiliations:** Department of Chemical Engineering, Imperial College London, LondonSW7 2AZ , U.K.

**Keywords:** porous media transport, convective mixing, dissolution trapping, spatial moment analysis, 4D X-ray computed tomography

## Abstract

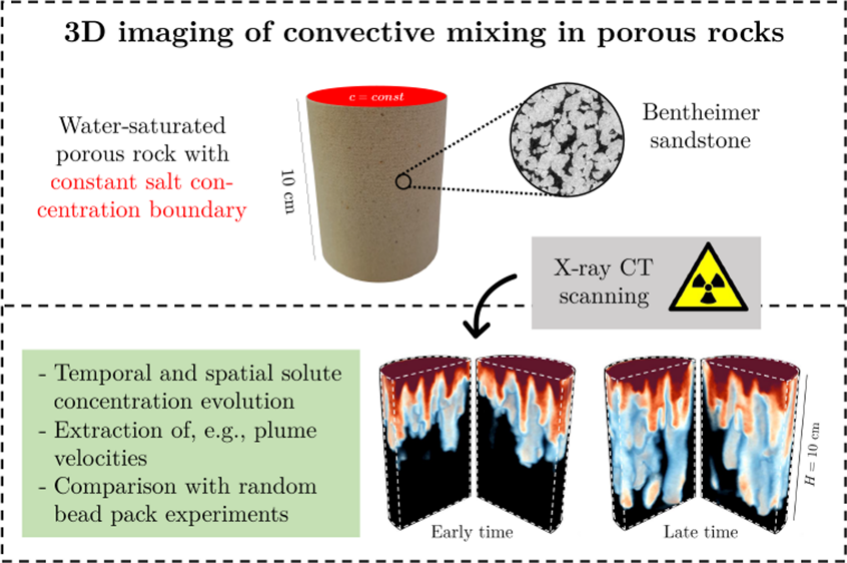

Free solutal convection refers to the mixing process induced and sustained by local density differences arising from solute dissolution. This process underpins the long-term storage of carbon dioxide (CO_2_) following its injection and dissolution in the formation brine of subsurface rock formations, such as saline aquifers. Direct experimental evidence of free solutal convection in porous rocks is to-date still lacking, leaving large uncertainties on the realized rate of CO_2_ dissolution and its contribution toward storage. Using an analogue solute–solvent pair and 4D X-ray computed tomography, we report direct observations of this mixing process in rock core samples, including sandstones and carbonates. The imagery is used to characterize the mixing structures that arise upon solute dissolution and to quantify differences between the rock types. Thus, we compute the temporal evolution of spatial moments of the concentration distribution to derive practical properties, such as the effective transport velocity of the solute plumes. Unlike previous studies on random bead packs, we observe that these measures do not scale well with core-scale rock properties (permeability, porosity, Rayleigh number) and are influenced by microscale rock characteristics (subcore and pore-scale heterogeneities). The latter may need consideration when evaluating the CO_2_ storage potential of candidate formations.

## Abstract 2

Free solutal convection refers to the mixing process induced and sustained by local density differences arising from solute dissolution. This process underpins the long-term storage of carbon dioxide (CO_2_) following its injection and dissolution in the formation brine of subsurface rock formations, such as saline aquifers. Unlike previous studies on random bead packs, we observe that measures of the convective mixing process do not scale well with core-scale rock properties and are influenced by both subcore- and pore-scale heterogeneities. The latter may need consideration when evaluating the CO_2_ storage potential of candidate formations.

## Introduction

The study of solute transport processes associated with fluid flow in natural porous media (e.g., rocks, sediments, soils) has become increasingly important for a wide range of environmental and industrial applications, such as soil contaminant remediation,^1,2^ nuclear waste repositories,^3,4^ enhanced oil recovery,^5,6^ and subsurface carbon dioxide (CO_2_) storage.^7^ The latter involves the injection of CO_2_ in depleted oil and gas reservoirs, coal seams, and saline aquifers, and is regarded as an effective technology to reduce continuous CO_2_ build-up in the atmosphere.^8^ Deep saline aquifers (e.g., permeable sandstone or carbonate formations) are suitable storage formations because they are distributed across the world, they hold the largest capacity, and they are saturated with brine, providing a means to sequester CO_2_ by dissolution.^9,10^ The mechanism by which CO_2_ dissolves into brine, which saturates porous rocks, is the subject of this study.

When CO_2_ dissolves into brine, it creates a solution that is denser than brine alone by an amount of up to 10 kg/m^3^,^11−13^ depending on salinity, pressure, and temperature. Because the dissolution process is initiated at the interface between the CO_2_-rich phase and the underlying aqueous phase, the diffusive boundary layer that is formed may become unstable as a result of local density differences. This instability can initiate motion in the fluids - a process referred to as *free* (or natural) solutal convection^14−16^ to highlight the role of gravity (as opposed to pressure) in sustaining flow. The following aspects of this convective process are worth emphasizing. The instability develops in the form of downwelling plumes, whose characteristic length scale depends on the balance between advection and diffusion.^17^ A key controlling parameter is the Rayleigh number, *Ra*, which defines the ratio between the characteristic time scales of these two transport processes.^18^ Specifically, free solutal convection can be sustained as long as the characteristic time scale for mass transfer via diffusion is much larger than that via advection. Important to storage security, the convective process promotes mixing between the CO_2_-rich solution and fresh brine, thereby increasing the rate of dissolution relative to a purely diffusive process.^18−20^

Free solutal convection has been studied extensively in the context of subsurface CO_2_ storage, both experimentally and computationally.^18^ In most cases, analogue porous systems have been considered, including transparent Hele-Shaw cells^21−30^ and unconsolidated packings of sand or beads. The latter has been studied as either uniform two-^31−36^ or three-dimensional systems.^37−51^ These studies have rationalized some of the fundamental scaling properties of the convective process and have unequivocally demonstrated that the free convective process enhances mixing.^43^ It was shown that the Sherwood number, a nondimensional measure of the convective mass flux, increases significantly with the strength of convection (i.e., with the Rayleigh number).^24,32,37,38,52^ Important to the application, the limitations of two-dimensional experiments (or their numerical counterparts) in deriving useful scaling laws have also been highlighted,^18,53−55^ promoting the use of 3D imaging technology in experiments^45^ and their validation by means of 3D numerical simulations.^17,56−61^

Despite a large number of contributions to the study of free solutal convection in porous media, direct experimental evidence of its occurrence in consolidated rocks is to-date still lacking. The multiscale heterogeneity of rocks is known to affect laboratory measurements of single- and two-phase flow properties, as demonstrated by advances in digital rock analysis in the last decades.^62,63^ In two-phase flow problems, the spatial variability in continuum properties (e.g., the capillary pressure curve) over length scales smaller than the typical laboratory rock sample introduces an apparent dependency of primary macroscopic observations (e.g., the fluid saturation^64^) on the rate and direction of flow.^65−67^ By analogy, solute transport in rocks is referred to as “anomalous” (or non-Fickian^68^) to indicate that macroscopic parameters (e.g., the dispersivity) depend on the time and/or length scale of measurement. Again, at the origin of this effect are macroscopic heterogeneities, i.e., regions with significantly different permeability values on a length scale of the order of millimeters.^69−72^ Our hypothesis here is that the presence of an external length scale in the problem (i.e., the characteristic heterogeneity scale of the rock sample) invalidates scaling laws of free solutal convection derived for uniform porous media. Initial experimental evidence in support of this hypothesis, albeit again for unconsolidated random packings and Hele–Shaw cells, indicates increased complexity of the mixing process as well as accelerated CO_2_ dissolution with the strength of spatial variations of permeability.^73,74^

To address the limitations of previous studies in representing subsurface systems, we conduct here an experimental investigation of free solutal convection in consolidated rock cores. A key novel contribution is the use of an imaging technique to provide direct experimental evidence of the occurrence of the convective process in both sandstone and carbonate systems. To this end, we use an analogue solute–solvent system that reproduces the linear increase of the density of water with the mass fraction of dissolved CO_2_ and deploy 4D X-ray CT to generate images of the solute concentration field at high spatial (mm) and temporal resolution (s). The experimental data set is the first of its kind and can inform 4D numerical studies of free solutal convection, which are just beginning.^75^ We qualitatively examine the evolution of individual plume structures in different rocks and quantitatively compare the mixing processes by computing the spatial moments of the solute concentration distribution. In this endeavor, we contrast the results with previous observations on unconsolidated beadpacks for the exact same analogue solute–solvent system.^37^ We further estimate measures of practical relevance, such as the average transport velocity of the sinking plume, to discuss the implications of our findings for the effectiveness of trapping by dissolution in subsurface CO_2_ storage.

## Methodology

This section describes the rock samples used in this study together with applied imaging techniques. Specifically, an X-ray micro-CT scanner is used for its ability to provide high-resolution imaging at the grain scale, useful for sample characterization, while a medical-grade X-ray CT scanner is used to enable dynamic imaging at the millimeter scale in real-time, necessary for capturing the details of the free solutal convective process.

### Rock Samples

**Figure 1:**
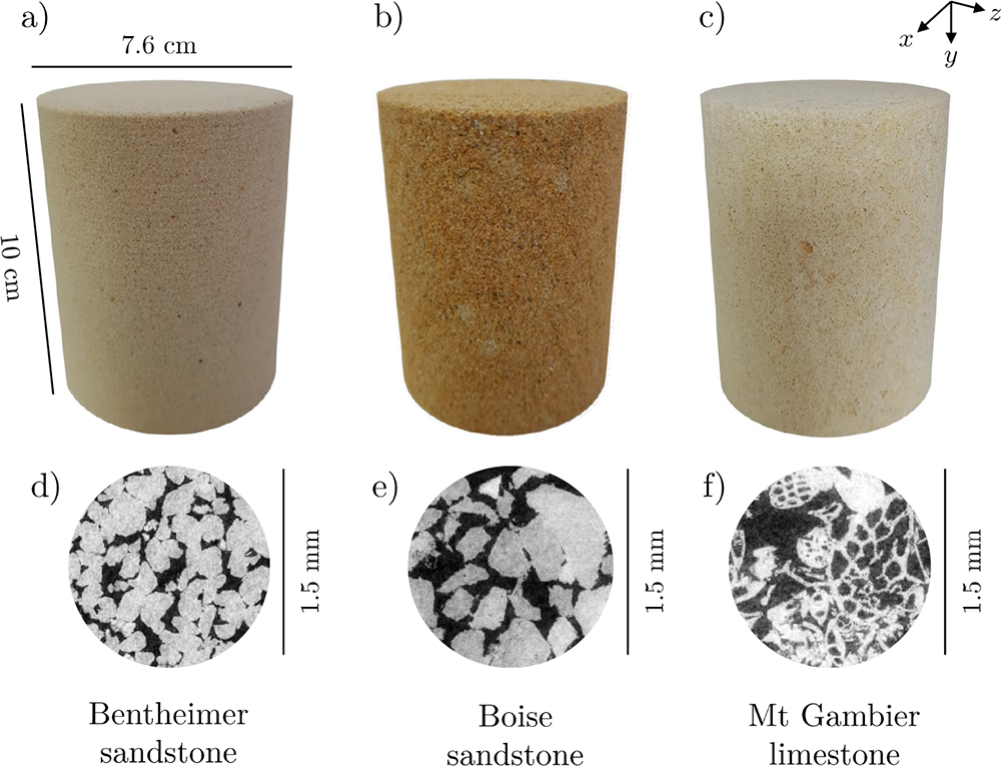
Rock samples used in this study. The upper panel of images shows digital photographs of the samples (a, Bentheimer sandstone; b, Boise sandstone; c, Mt Gambier). The lower panel of images shows two-dimensional gray-scale X-ray tomograms obtained by high-resolution micro-CT (the voxel size of the images is 3.9 μm).

The rocks used in this study are Bentheimer sandstone, Boise sandstone, and Mount Gambier limestone (in the following referred to as Mt Gambier) and they have been sourced from Kocurek Industries INC (Caldwell, TX, USA). Digital photographs of the 7.6 cm-diameter and 10 cm-long samples used in the experiments are shown in Figure 1. The samples have been selected to represent a range of reservoir properties and rock types, including structures that are typical of clastic rocks (e.g., the vertical layering visible in Boise sandstone, shown in the Supporting Information (SI) in Figure S1) and carbonate sedimentary formations (e.g., the heterogeneous bulk density distribution observed in Mt Gambier, Figure S1). The structurally uniform Bentheimer sandstone was used because of its utility in making comparisons with the more heterogeneous rocks. Figure 1 also shows representative grayscale X-ray tomograms acquired by microcomputed tomography (Thermo Fisher Heliscan micro-CT scanner, voxel size 3.9 μm) on dry 1.5 cm-diameter subsamples cored from a sister plug. Relevant petrophysical properties of the rock samples are summarized in Table 1, and include the porosity (total, ϕ_t_ and mobile, ϕ), the characteristic length-scale (*L*_c_), the permeability (*k*), and the tortuosity (τ). The description of the methods used to estimate these properties is presented in Texts S1–S5 (see also caption of Table 1), including the analysis of the representative elementary volume (REV) for each rock sample.

**Table 1:** Relevant Petrophysical Properties of the Rock Samples (Uncertainties Are Reported in Brackets)^a^

rock sample	Bentheimer	Boise	Mt Gambier
total porosity, ϕ_t_ [−]	0.21 (0.01)	0.28 (0.01)	0.51 (0.01)
mobile porosity, ϕ [−]	0.22 (0.01)	0.26 (0.01)	0.51 (0.01)
char. length-scale, *L*_c_ [μm]	260 (3)	249 (23)	150 (14)
permeability, *k* [Darcy]	1.42 (0.01)	4.48 (0.01)	12.99 (0.07)
tortuosity, τ [−]	3.9 (0.2)	3.2 (0.5)	3.5 (0.3)
buoyancy velocity, *u*_B_(Δρ_max_) [cm/min]	0.06 (0.01)	0.18 (0.02)	0.54 (0.01)
Rayleigh number, *Ra* [−]	6600 (400)	14,700 (2500)	24,300 (2400)

a The total porosity ϕ_t_ is measured with a medical X-ray CT scanner (Toshiba Aquilion TSX-101A) by differential imaging (dry-water scans). Binarised high-resolution X-ray images (micro-CT scanner, Thermo Fisher Heliscan) are used to obtain estimates of the mobile porosity, ϕ, the pore-space tortuosity, τ, and of the characteristic length-scale, *L*_c_ = *V*/*S*, where *V* is the imaged bulk volume of the sample and *S* is the pore-solid interface area (see SI Text S3). The permeability *k* is estimated from the application of Darcy’s law to steady-state multi-rate flow tests with brine (see SI Text S5). The estimation of the buoyancy velocity *u*_B_ (eq 1) and the Rayleigh number *Ra* (eq 2) is described in the text.

**Figure 2:**
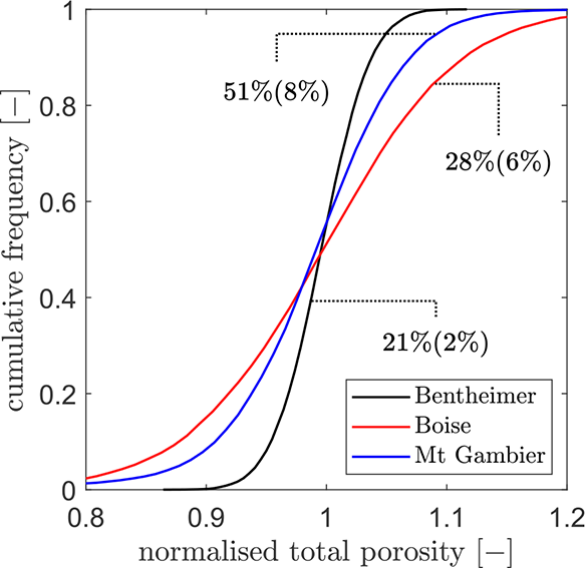
Cumulative distribution of the subcore scale porosity of the rock samples used in the free solutal convection experiments. The porosity distribution is obtained by analyzing 3D tomograms acquired by X-ray CT reconstructed with a voxel size of (3 × 3 × 0.5) mm^3^ for a subset of the sample of volume (30 × 30 × 95.5) mm^3^. The porosity values are normalized by the mean sample porosity, ϕ_t_, indicated in the plot together with the absolute standard deviation of the distribution.

Bentheimer sandstone is a well-sorted (*L*_c_ = 260 ± 2 μm) sedimentary rock with good permeability (*k* = 1.4 Darcy) and moderate porosity (ϕ_t_ = 21%). It mainly consists of quartz (around 99 wt %), and is often regarded as a useful benchmark due to its uniform texture and simple chemical makeup.^76,77^ Boise sandstone has a less uniform texture (*L*_c_ = 249 ± 16 μm), is rich in quartz (around 40 wt %) and feldspar (around 50 wt %^78^), and has much higher permeability (*k* = 4.5 Darcy) and porosity (ϕ_t_ = 28%). Mt Gambier is a highly porous (ϕ_t_ = 51%) and highly permeable (*k* = 13 Darcy) fossiliferous limestone. Compared to the two sandstones, its pore-space is highly heterogeneous with visible structures made of fossil shells and bryozoae (*L*_c_ = 150 ± 8 μm).^79^ For all rock samples, the total (ϕ_t_) and mobile porosity (ϕ) are very similar, indicating that the porosity is found above the resolution of the microCT scanner and is thus likely accessible to flow. The pore-space of the three rocks also presents a similar average tortuosity, i.e., 3 < τ < 4. However, as shown in Figure 2, the core samples used for the convective mixing experiments present noticeable differences in their porosity distribution at the mm-scale. Specifically, while the porosity in Bentheimer sandstone remains very uniform (2% deviation from the mean), broader distributions are observed for Boise sandstone and Mt Gambier (6 and 8% deviation from the mean, respectively).

### Free Solutal Convection Experiments

In Eckel et al.,^37^ we have presented the development of an image-based experimental protocol that uses X-ray CT technology to visualize the temporal and spatial evolution of convective mixing within a porous medium. The method uses potassium iodide (KI) as a contrast agent which, upon dissolution into the water that saturates the pore space of the rock sample, triggers the convective flow. As such, the aqueous KI solution is used in this study to represent brine saturated with dissolved CO_2_, to transfer our findings to free solutal convection in CO_2_ storage applications. The following important properties of the surrogate fluid pair, aqueous KI solution, and water, are noted:

- Similar to the CO_2_-brine system and for the conditions relevant to subsurface CO_2_ storage,^11^ the density of the aqueous KI solution increases monotonically and approximately linearly with the solute mass fraction, *w*.^37^
- The maximum density of the solution at maximum solubility is ρ_max_ = 1718 kg/m^3^ (*w*_max_ = 0.6 kg/kg, *T* = 298.15 K).^80^ The difference with pure water (*w* = 0, ρ_w_ = 997 kg/m^3^ at *T* = 298.15 K) is large enough to drive convection in the fluid underneath the interface even for media with relatively low permeability (such as natural rocks).
- The change of the solution viscosity with mass fraction at *T* = 298.15 K is significantly smaller than the change in the solution density over the entire range of solute mass fraction, an average relative change of approximately 2 and 40% for the viscosity and the density, respectively.^81^ Therefore, in this study, the viscosity of the solution is regarded as constant and independent of the solute mass fraction.
- The X-ray mass attenuation coefficient of the saturated solution is roughly four times higher than the one of pure water, leading to high contrast in the CT images.^82^

In the design and interpretation of the experiments, the following quantities and dimensionless groups have been considered. We define a buoyancy velocity scale, *u*_B_,^55^
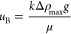
1where Δρ_max_ = ρ_max_ – ρ_w_ = 721 kg m^–3^, the viscosity of pure water at ambient conditions is μ = 1.002 mPa s, and the acceleration due to gravity is *g* = 9.81 m s^–2^. The velocity scale is used to define the dimensionless time, *t̃* = *t*/*t*_B_ = *tu*_B_/*H*, which has been found to correctly scale-free solutal convection experiments on homogeneous and uniform glass bead packs (SI Figure S7). The velocity scale also appears in the definition of the Rayleigh number, *Ra*(15):
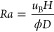
2where *H* = 10 cm is the sample height and *D* is the effective diffusion coefficient in the porous medium (*D* = *D*_m_/τ), where *D*_m_ = 2.61 × 10^–9^ m^2^ s^–1^ is the molecular bulk diffusion coefficient of aqueous KI-solution^83^ (SI Text S8). Values of *u*_B_ and *Ra* estimated for the experiments reported in this study are listed in Table 1. In making these estimates, we have accounted for uncertainties in the measured permeability, porosity, and tortuosity (SI Texts S1, S5, S7) through the application of classic rules of error propagation. Despite the presence of moderate uncertainties, the samples indicate a clear trend of increasing *Ra* in the order Bentheimer < Boise < Mt Gambier. We note that the buoyancy velocity scale, *u*_B_, represents the maximum possible velocity that is likely to occur only near the interface. In interpreting the experiments, we will also consider an average transport velocity, *u*_y,av_ (and its associated value of *Ra*), which can be extracted from the average displacement of the center of mass of the solute plume (see Discussion section).

### Sample Preparation and Experimental Procedure

**Figure 3:**
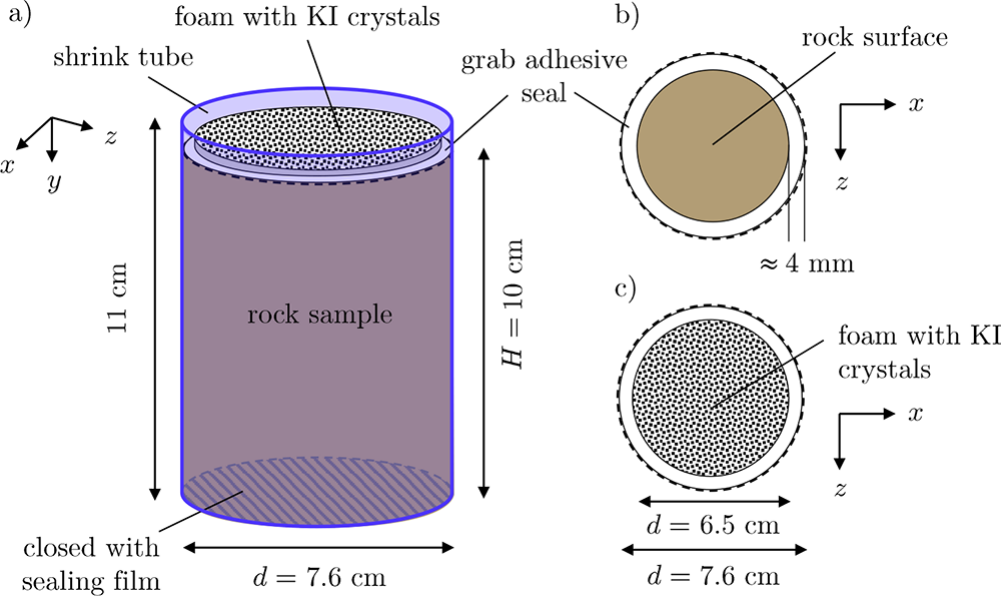
Drawing of the prepared rock sample used for the free solutal convection experiments. (a) View from the side. (b) View from the top before the start of the experiment. (c) View from the top during the experiment.

A schematic representation of the experimental setup used for the free solutal convection experiments is shown in Figure 3. Ahead of each experiment, the sample must be saturated with tap water. To this end, the cylindrical rock core is placed between two end-caps equipped with o-rings and it is wrapped with an 85 mm-diameter polyolefin heat shrink tube (TE Connectivity, Switzerland). The shrink tube is lined with adhesive, which melts upon heating with a heat gun to achieve a tight seal. The rock core is then purged with gaseous CO_2_ (purity <99%, BOC Ltd., UK) to displace air from the pore space, followed by the injection of 7 pore volumes of tap water using a syringe pump (Teledyne ISCO, Model 500D) to achieve full saturation.

After the end-caps are removed, the shrink tube is cut flush at the bottom end of the sample, while a 1 cm margin is left at the top end. Two layers of sealing film (Parafilm M) are applied to close the bottom end of the sample and fixed in place with waterproof tape. To maintain complete saturation, the sample is submerged in tap water for 24 h. While keeping the sample submerged, waterproof grab adhesive (Heavy-Duty Grab Adhesive, Gorilla Glue, USA) is applied with a small spatula around the perimeter of the open face of the sample and left curing for at least 3 h. This roughly 4 mm wide layer creates a seal between the shrink tube and the rock core to prevent flow along the rock-tube interface, where permeability is inevitably higher. The sample is removed from the water and placed in a vertical position in the gantry of the medical-grade X-ray CT scanner (Toshiba Aquilion TSX-101A).

A thin round-shaped melamine resin foam (6.5 cm radius, 0.5 cm thickness, model Basotect, supplied by BASF SE, Germany) is soaked in saturated KI solution and a roughly 2 mm thick layer of KI salt is spread on top. To initiate the experiment, the foam is quickly, yet carefully, positioned on top of the rock sample using a broad spatula by allowing it to slide smoothly across the entire surface and taking care to avoid trapping any air bubbles between the foam and the rock surface. The foam has a much higher porosity (>90%) than the rocks and it has a relatively low permeability. The high porosity yields liquid-like properties to the foam, while the low permeability minimizes any upward movement of low-density plumes during the experiment. The crystalline KI on top of the foam is finely grained, allowing it to dissolve readily in water by diffusion. The foam is also very thin, effectively creating a layer of constant solute concentration and maximum solubility at the top boundary of the rock. The excellent reproducibility of experiments carried out on each rock sample indicates that the procedure yields a consistent onset time of convection.

Tomograms of the entire rock sample are taken at regular intervals during the experiment by setting the peak potential of the X-ray tube to 120 kVp and the tube current to 200 mA. One full tomogram is acquired in helical mode in just a few seconds. When considering the estimates of the maximum buoyancy velocity, *u*_B_ (Δρ_max_), reported in Table 1, a linear displacement of the solute plume of at most 0.5 mm in 5 s is achieved, corresponding to 0.5% of the length of the sample. Therefore, upon application of the proposed scanning protocol, it is plausible to assume that the solute plumes do not move during the acquisition of one full tomogram. During the initial phase of the experiment, scans are taken every few seconds and their frequency is reduced to a few minutes during the final phase of the experiment. The experiment ends once the solute plumes reach the bottom of the sample. To maintain saturation concentration in the foam throughout the experiment, more crystalline KI is supplied by sieving it on top carefully between scans. The experiment is repeated twice for each rock type by using two different core samples, referred to as sample #1 and sample #2 in the following. The duration of the experiment varied depending on the rock type–lasting approximately 1 h for Bentheimer, and approximately 30 min for Boise and Mt Gambier.

### Image and Noise Analysis

The X-ray CT scanner applies a three-dimensional quantum denoising filter to reconstruct images in Hounsfield units (HU) at a spatial resolution of 1 mm in the *z-*direction and 0.2 mm in the *x-* and *y*-directions. For image analysis, the reconstructed images of the 10 cm long rock samples are cropped 13 mm from the foam-rock interface and roughly 15 mm from the outer boundary of the sample, to avoid imaging artifacts and reduce the effects of wall boundaries in the calculations. Details on the conversion of the images from HU units to solute mass fraction *w* and solute molar concentration *c* are reported in Eckel et al.,^37^ together with the derivation of the equations used to compute the spatial moments of the solute concentration field. For the sake of clarity, only the relevant equations are summarized in the following. All of the analysis was carried out using in-house MATLAB routines.

The solute molar concentration at each voxel location *i* is computed as
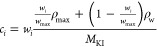
3where *M*_KI_ = 0.166 kg mol^–1^ is the molar mass of the solute and the solute mass fraction *w*_*i*_ is obtained directly upon a suitable combination of the X-ray CT tomograms:
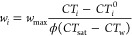
4where *CT*_*i*_ is the CT number in HU of voxel *i* for a given scan and *CT*_*i*_^0^ is the value of the same voxel at the beginning of the experiment. *CT*_sat_ = 9505 HU and *CT*_w_ = 40 HU are the constant CT numbers of the KI-saturated solution and of pure water, respectively, obtained from a previous calibration. In applying eq 4, the assumption is made that the porosity ϕ is constant and independent of the spatial location. Therefore, during the experiments, the solute molar concentration varies between maximum concentration (*w* = *w*_max_ in eq 3, yielding *c* = 6198 mol/m^3^) and pure water (*w* = 0 in eq 3, yielding *c* = 0 mol/m^3^), as a result of the mixing process.

To analyze the free solutal convective process, we compute the one-dimensional vertical solute concentration function *c*_*y*_(*y*) by averaging the local concentration values *c*_*i*_ for each *x*–*z* horizontal slice along the *y*-direction. This quantity is then used to compute the first three spatial moments of the concentration distribution, *m*_*j*_ (*j* = 0, 1, 2), as given by the following general expression:
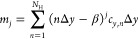
5where the analyzed domain has been discretized into *N*_H_ slices of thickness Δ*y* and *c*_*y*,*n*_ is *c*_*y*_ computed at *y* = *n*·Δ*y*. We also note that β = 0 for *j* = 0 and *j* = 1, and β = *m*_1_/*m*_0_ for *j* = 2. Therefore, the total mass of solute in the domain is obtained as *M* = *m*_0_*A*ϕ, where *A* is the cross-section of the analyzed domain. The location of the center of mass in the longitudinal direction is given by *Y*_com_ = *m*_1_/*m*_0_, while the standard deviation of the solute concentration distribution is obtained as .

As discussed in our previous publication,^37^ the noise observed in the CT images is mainly due to quantum mottle (i.e., random imaging noise). The uncertainty of the CT number was estimated using repeated scans of the saturated rocks taken prior to the start of the experiment and amounts to σ_Δ_CT__ ≈ 17 HU for voxels of size (0.26 × 0.26 × 1) mm^3^. Using statistical methods of error propagation (SI Text S6), the uncertainties of the computed solute mass fraction increase with solute concentration in the range σ_*w_i_*_ = 0.01–0.09 for *w*_*i*_ = 0–0.6. Accordingly, the uncertainty in vertical concentration distribution is estimated to vary σ_c_*y*,*n*__ = 46–1421 mol/m^3^ for *c*_*y*,*n*_ = 0–6198 mol/m^3^.

## Results

### Three-Dimensional Convective Pattern

**Figure 4:**
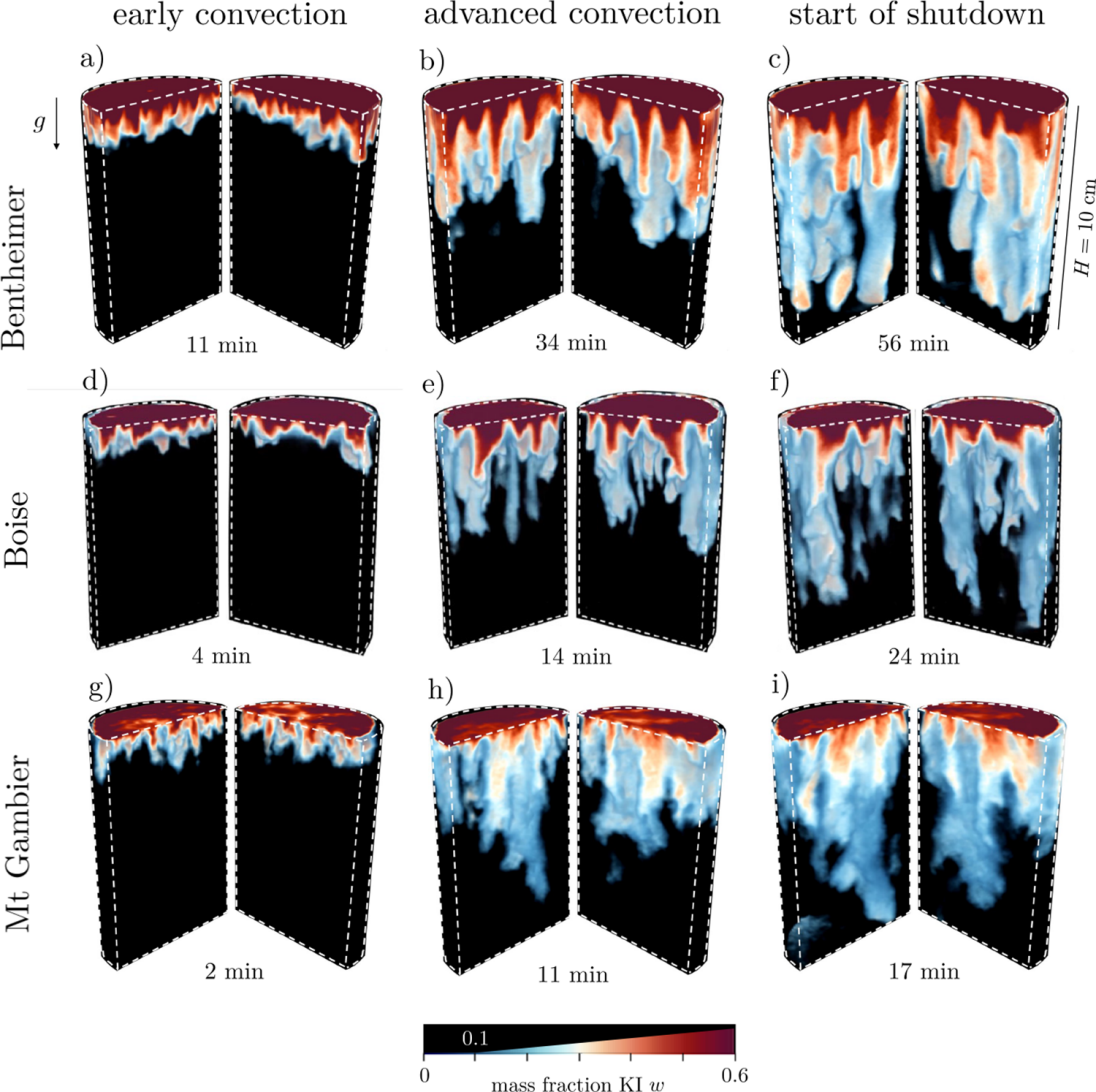
Three-dimensional reconstructions of the solute mass fraction field at different times for Bentheimer sandstone (a–c), Boise sandstone (d–f), and Mt Gambier (g–i). The reconstructions are cut vertically in two halves for better visualization. The times indicated for the advanced convective regime and the start of shutdown are chosen as the times when the first plumes reach *y* = *H*/2 and *y* = *H*, respectively. Pure and very low concentrated water (*w* < 0.1) is made transparent (background is black) and the intensity of the color bar is gradually increasing between 0.1 < *w* < 0.6. The results refer to experiments with sample numbers Bentheimer #1, Boise #2, and Mt Gambier #2.

Snapshots of the free solutal convective process are shown in Figure 4 for each rock sample at three distinct times to represent the three main convective regimes (early convection, advanced convection, and start of shutdown). It can be seen that the mixing of the KI-saturated solution with the water near the rock-foam interface generates a denser solution that leads to the formation of dissolution plumes. These images clearly demonstrate the occurrence of free solutal convection in porous rocks. Yet, distinct dissolution patterns are observed for the three rocks as a result of the different *Ra* regimes as well as their distinct subcore and pore-scale properties. The downwelling plumes in Bentheimer sandstone are columnlike, evenly distributed across the width of the sample, and retain large solute concentrations to greater depths relative to the other samples. In Boise sandstone, the number of plumes is smaller than that in Bentheimer sandstone. This is contrary to expectations from experiments in Hele-Shaw cells^84^ or with unconsolidated bead- or sand-packs,^37,42,85,86^ where a larger value of *Ra* translated into a larger number of plumes. Also notable is the reduction in the number of plumes as a result of their merging in the advanced convection regime. In Mt Gambier, the dissolution structures forming out of the initial instability appear frayed without clear contours. In the advanced convective regime, one can no longer identify the columnar-like pattern observed in the sandstones. Rather, the solute is transported within a few large clouds with a substantial dilution of concentration gradients.

**Figure 5:**
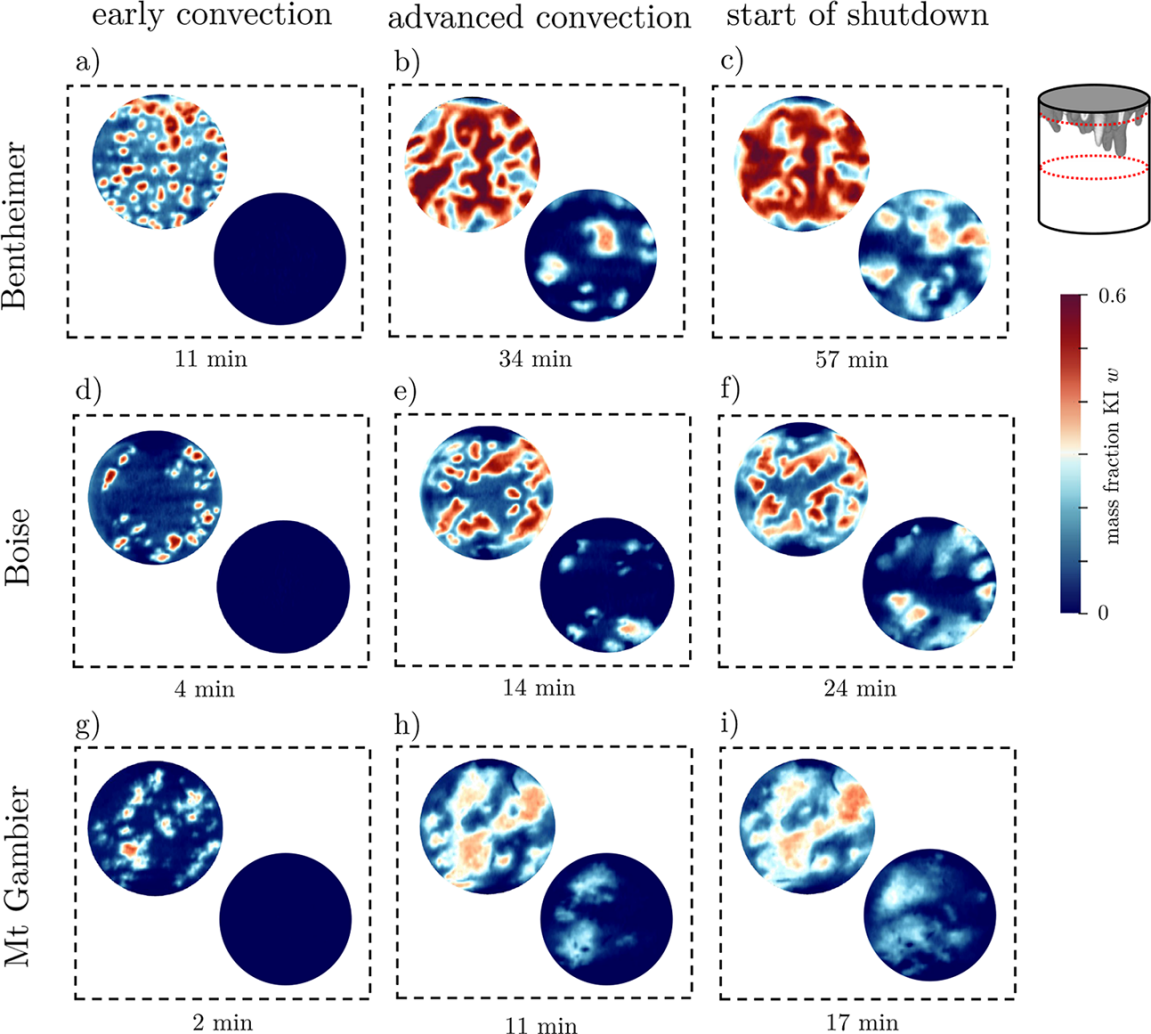
Two-dimensional maps of the solute mass fraction field at different times for Bentheimer sandstone (a–c), Boise sandstone (d–f), and Mt Gambier (g–i). Two locations are shown, namely, 50 voxels (*y* ≈ 86 mm) below the rock-foam interface (top left in each panel) and at *y* = *H*/2. The same times as in Figure 4 are shown. The results refer to experiments with sample numbers Bentheimer #1, Boise #2, and Mt Gambier #2.

The same images discussed above are presented in Figure 5 as 2D maps representing the sample cross-section just below the rock-foam interface and midway along the sample. We can see that in Bentheimer sandstone many small plumes are initially formed relative to Boise sandstone and Mt Gambier. In Bentheimer sandstone, as time goes by, the merging of plumes forms a complex maze-like pattern of highly concentrated solute. This characteristic self-organization of the downwelling plumes has been observed in previous experimental and numerical work with unconsolidated bead packs,^38,75^ but has not been experimentally verified in rocks until now. The maze-like pattern is not observed in Boise sandstone and Mt Gambier, where the network is poorly connected and shows strong dilution for the limestone sample. Detached columnar plumes are still observed midway through the sample in Bentheimer and Boise sandstones; however, their number is reduced relative to the number generated near the interface, indicating that the merging of smaller plumes occurs as they elongate. On the contrary, for Mt Gambier, it is not possible to make out contours and to distinguish individual plume bodies at late times.

### One-Dimensional Concentration Profiles

**Figure 6:**
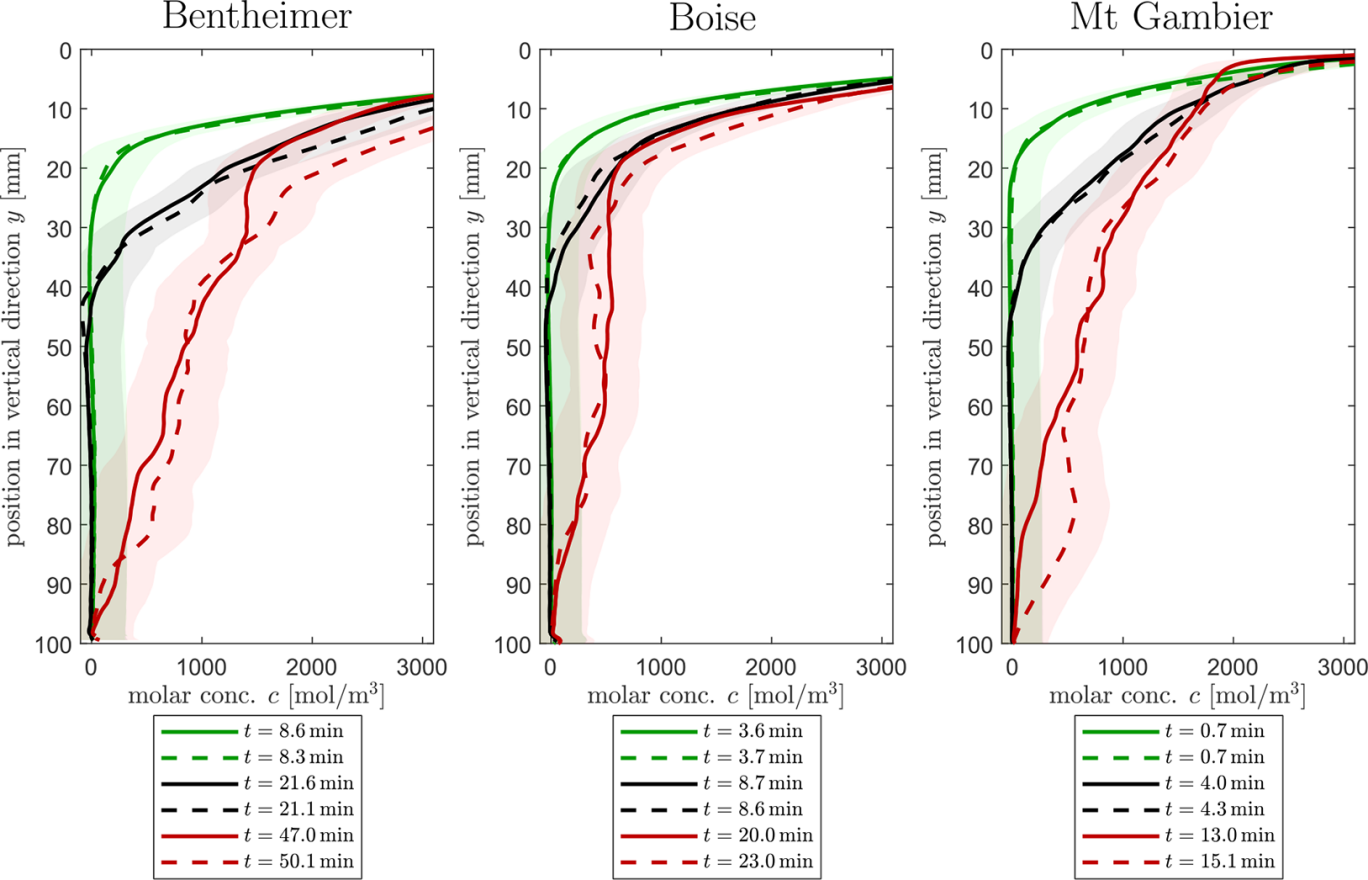
Horizontally averaged concentration profiles *c*_y,*n*_ along the position in the vertical direction (*y*). The solid and dashed lines represent the data of the two sister rock samples #1 and #2, respectively. For each rock sample, the profile is shown at three different times (early convection: first plume reaches *y* = *H* – 13 mm, advanced convection: first plume reaches *y* = *H*/2, start of shutdown: first plume reaches *y* = *H*). The color-shaded area represents the uncertainty of *c*_y,*n*_ which is computed using statistical methods of error propagation to eq 3. For simplicity, the uncertainty calculations for all error bars utilized the average concentration value across the entire length of the rock. The uncertainty calculations also incorporate an average value between the mass fraction’s minimum and maximum expected uncertainty (σ_*w_i_*_ = 0.04).

To quantify the observations in Figures 4 and 5, we plot the horizontally averaged solute concentration over depth at various times (Figure 6). For each rock sample, we consider the profiles at three distinct times–representative of early convection, advanced convection, and the start of shutdown (see caption for additional details). We observe excellent agreement between experiments carried out on the two sister samples of each rock type (dashed and solid lines). Importantly, the profiles of the three rock samples are distinctly different, indicating that the convective mixing phenomenon evolves differently even when the dimensionality of the system is reduced. Specifically, we note that for each rock the three concentration profiles overlap up to a specific location, namely *y* ≈ 10 mm for Bentheimer sandstone, *y* ≈ 5 mm for Boise sandstone, *y* ≈ 2 mm for Mt Gambier. We interpret this characteristic depth as the point beyond which the solute plumes originating at the rock-foam interface are affected by the strong dispersion associated with the convective process. Because the concentration prior to this characteristic depth is uniform, we thus hypothesize that the latter is an indicator of the strength of subcore scale heterogeneity, i.e., the smaller the value the stronger the heterogeneity and the stronger the dispersive process. Accordingly, we observe that the uniform texture of Bentheimer sandstone enables reaching much higher solute concentrations at greater depths (*c* ≈ 1000 mol m^–3^ at *y* = 50 mm) relative to those of the other two rocks (*c* ≈ 500 mol m^–3^ at *y* = 50 mm) by the time the first plume reaches the bottom of the sample.

### Spatial Moments of the Solute Mass

**Figure 7:**
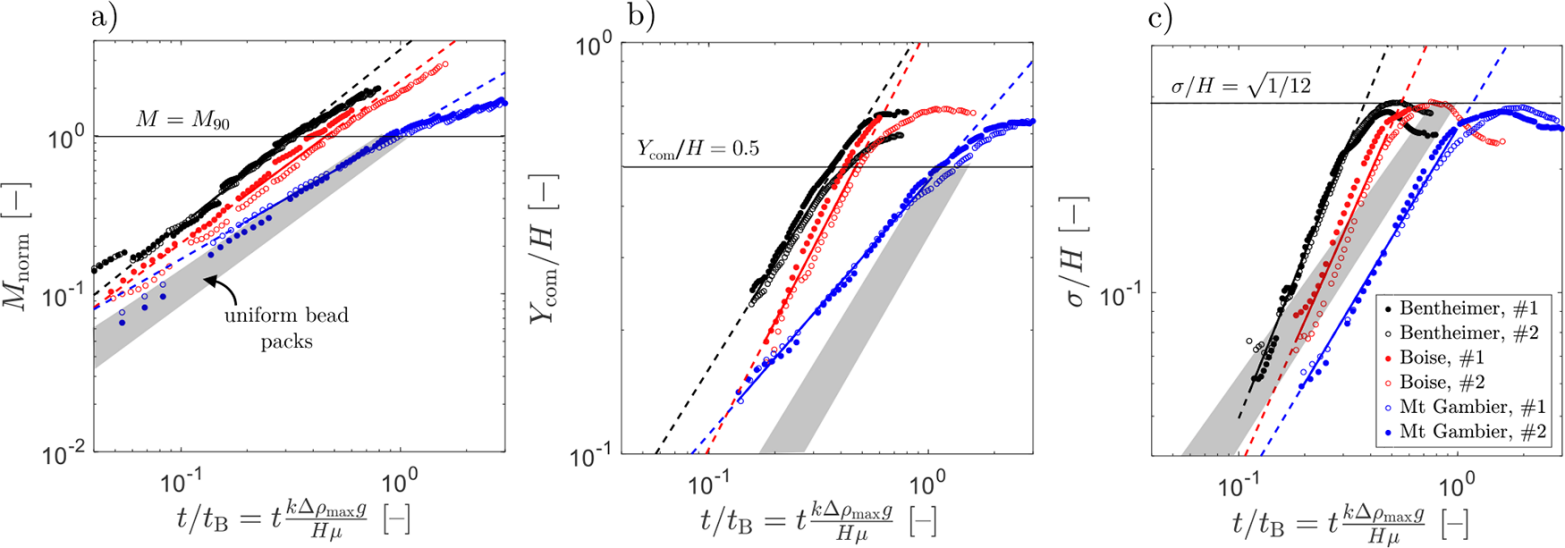
Spatial moments of the solute mass plotted as a function of normalized time *t̃* = *t*/*t*_B_ for the three rock samples. (a) Zeroth moment represented as normalized total mass, *M*_norm_ = (*M*(*t*) – *M*_0_)/(*M*_90_ – *M*_0_). *M*_0_ and *M*_90_ refer to the start of the experiment and the attainment of near-complete mixing (i.e., σ = 0.9σ_max_, where  and *Y*_norm_ = *H*/2), respectively. (b) First moment represented as the dimensionless vertical center of mass *Y*_norm_/*H*, where *H* is the sample height. (c) Second moment represented as the dimensionless standard deviation of the solute concentration distribution around the mean in the vertical direction, σ/*H*. Empty and filled symbols refer to experiments conducted on different samples of the same rock type. The solid lines (color-coded) are power-law fits to the experimental data in the convective regime (the line is dashed outside this regime). The gray-shaded area refers to results of experiments conducted using random beadpacks (*Ra* = 3000–55,000 from Eckel et al.^37^).

The analysis of the spatial moments of the solute mass and their evolution over time can aid the quantitative analysis of the free solutal convective process. The (a) total mass of the solute (*M*_norm_), (b) the location of the center of mass in the *y*-direction (*Y*_norm_/*H*), and (c) the standard deviation of the solute concentration distribution (σ/*H*) are plotted as a function of the dimensionless time *t̃* = *t*/*t*_B_ in Figure 7 for the three rock samples. The same data plotted as a function of the absolute time, *t*, are presented in the SI (Figure S8). In each plot, the gray-shaded area refers to the results of experiments conducted using random beadpacks (distinct bead sizes to yield *Ra*(*u*_b_) = 3000–55,000).^37^ We note that three measures, computed for the three different beadpacks, scale with *t̃*, as the dependence on permeability is effectively removed (see SI Figure S7 for additional details). On the contrary, stark differences are observed between rock types, especially between the two sandstones and the carbonate, the results for the latter being systematically shifted toward larger values of *t̃*. The fact that each individual experiment shows excellent reproducibility (empty and filled symbols) for all spatial moments and for each rock type suggests that other properties affect the mixing process.

For each rock type, the temporal evolution of the three measures of the mixing process in the convective regime follows a power-law behavior, i.e., , , σ/*H* ∝ *t̃*^*b*_3_^. The convective regime starts approximately at *t̃* = 0.1–0.2 and ends when σ = 0.9σ_max_ which is approximately the time when the solute reaches the bottom of the sample (see the caption of Figure 7). It is worth noting that the experiments have been interrupted before reaching complete mixing (*Y*_com_ = *H*/2 and , respectively). Pooling at the bottom of the domain causes *Y*_com_ to grow beyond this threshold, which would eventually approach asymptotically from above. Similarly, the pooling causes a decrease in σ, which is therefore expected to grow upon additional mixing. The power-law exponents *b* for each rock type and spatial moment are summarized in Table 2 together with the 95% confidence intervals. The evolution of the zeroth moment for the two sandstones follows a ballistic power-law behavior (*b* ≈ 1). In agreement with observations on glass bead packs,^37^ nearly linear growth of the mass of dissolved solutes is an indication of attaining a constant dissolution flux. On the contrary, for Mt Gambier the power-law exponent of the zeroth moment is less than 1 (*b* = 0.81 [0.78, 0.84]). This observation further indicates that the dissolution rate is reduced and decreases over time. In Figure 7a, we have used the system height *H* as the characteristic length scale in the definition of the characteristic time scale, *t*_B_. We note that *H* would become relevant only once the fingers reach the bottom of the sample. As such, it could be useful to use a so-called diffusive length scale, *L* = ϕ*D*/*u*_B_. We provide a comparison of Figure 7a by using these different length scales in Figure S9. We note that using a different length scale would not change the quantitative interpretation of the results in Figure 7a, as the power-law coefficient would not change upon switching the length scale.

**Table 2:** Exponents *b* of the Power Law Fittings () Determined in the Advanced Convective Regime for the Moments of Solute Mass^a^

sample	Bentheimer	Boise	Mt Gambier	glass beads
zeroth moment	1.11 [1.08, 1.15]	1.02 [0.89, 1.16]	0.81 [0.78, 0.84]	0.96–1.05
first moment	0.86 [0.82, 0.90]	1.03 [0.93, 1.12]	0.63 [0.61, 0.64]	0.86–0.92
second moment	1.34 [1.30, 1.38]	1.21 [1.10, 1.32]	0.89 [0.85, 0.93]	0.74–0.84

a The 95% confidence bounds for the fitted exponents are given in brackets.

The analysis of the evolution of the first spatial moment supports these early indications that the convective mixing process in the carbonate sample is fundamentally different from that in the sandstones. For Bentheimer, the power-law exponent (*b* = 0.86 [0.82, 0.90]) agrees well with observations on bead packs (*b* = 0.86–0.92, see Eckel et al.^37^). While for Boise a larger exponent is obtained (*b* = 1.03 [0.93, 1.12]), the latter is still near ballistic scaling. On the contrary, the value obtained for Mt Gambier is significantly smaller (*b* = 0.63 [0.61, 0.64]), indicating a slower downward movement of the plume with respect to the normalized time. This result is quite noteworthy given that the permeability of Mt Gambier is substantially larger than the permeability of the two sandstones (see Table 1).

The second spatial moment is often interpreted as the spreading length^87,88^ – representing here the characteristic distance in the *y*-direction over which the solute plume spreads around the center of mass. For a purely diffusive process, this measure is expected to follow the power-law scaling, σ/*H* ∝ *t̃*^0.5^. As expected, for all three rocks, the power-law exponent *b* > 0.5. The two sandstones show unexpectedly high exponents (*b* = 1.2–1.3), relative to the benchmark glass beadpacks (*b* = 0.74–0.84, depending on the glass bead size). While the two porous systems share a similar permeability (approximately 1.5 Darcy for random packing of beads with a diameter of 55 μm), they differ in their porosity (ϕ ≈ 0.22–0.26 for the rocks vs ϕ ≈ 0.34–0.42 for beadpacks). We thus hypothesize that the larger spreading in the sandstones is the result of a reduced extent of (transverse) mixing during convection. In the three-dimensional convective patterns, we note, in fact, that for the sandstones, the solute concentration within the downwelling plumes remains quite high because less pore volume is available for solute dissolution. For the carbonate rock, we observe *b* = 0.89. This rock has a much higher permeability and porosity compared to the other systems (sandstones and bead packs). As permeability enhances spreading while porosity favors mixing, we conclude that the similarity of the power-law exponent of Mt Gambier with the estimate obtained for beadpacks is only coincidental and the result of these two opposing effects.

## Discussion

**Figure 8:**
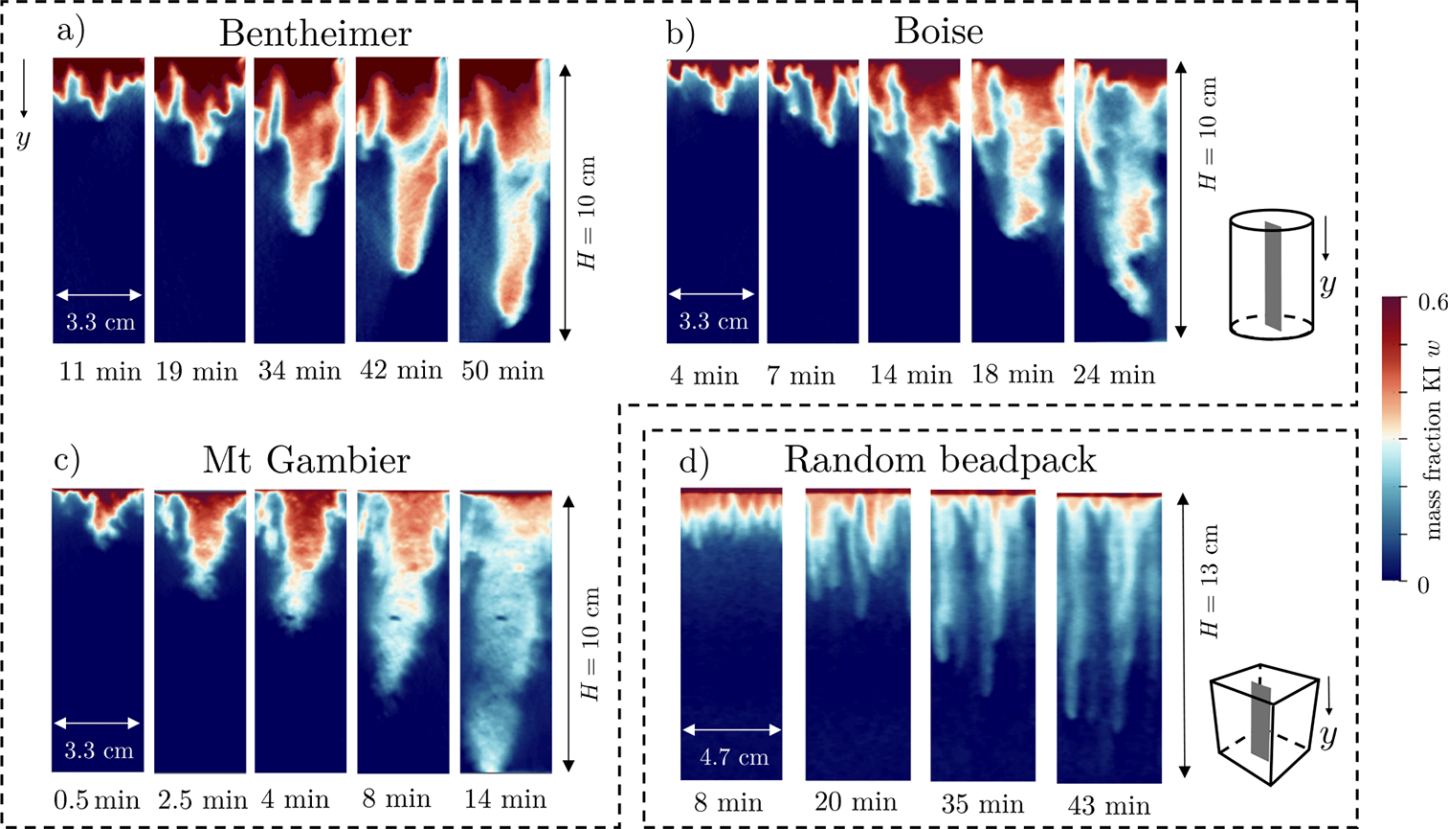
Evolution of discrete solute plumes over time for (a) Bentheimer #1, (b) Boise #2, (c) Mt Gambier #2, and (d) a random bead pack with bead size of 100 μm (data from Eckel et al.^37^). Each panel shows a two-dimensional section taken from the central plane of the 3D reconstructions, as illustrated for the rock samples (panels a–c) by the sketch of a cylinder in the top right corner and for the bead packs (panel d) by the sketch of a cube in the bottom right corner.

The results presented thus far indicate that the free solutal convective process observed in rocks differs substantially from observations made with unconsolidated porous media, including results reported in our previous study on uniform bead packs.^37^ In bead packs, changes in the plume structures were commensurate with changes in *Ra*: broadly similar columnar plumes were observed among experiments conducted with different bead sizes, with the only differences being the number of plumes (increasing with *Ra*) and their width (decreasing with *Ra*). In rocks, the opposite trend is observed (the number of plumes decreases with increasing *Ra*) and the plume structures can deviate substantially from the columnar shape. Because the experiments show excellent reproducibility for all spatial moments and for each rock type, we hypothesized that rock-specific textural properties are a controlling factor. To support this argument, we show in Figure 8 the evolution of discrete solute plumes for the three rocks (panels a, b, and c) and for one exemplary experiment with a random beadpack using the same fluid pair and a similar experimental protocol (panel d, from (37)). The images indicate two effects: (1) stronger longitudinal spreading in the sandstone samples relative to the beads and the carbonate sample and (2) stronger lateral mixing in the carbonate sample relative to the bead pack. With regard to (1), we note that in Boise and, to a greater extent, in Bentheimer the plumes retain a larger concentration to greater depths than Mt Gambier and the random beadpack, indicating that in the sandstone the transport process shows limited dispersion and mixing. With regard to (2) we note that the plumes in the carbonate sample and the random beadpack become strongly diluted. Yet, although diluted, individual columnar plumes are still visible in the random beadpack up to late times, as opposed to Mt Gambier, where a large diluted plume is formed from the outset. These direct observations confirm that the complex texture of rocks controls the convective pattern and the resulting concentration field, with potential implications on the realized rate of CO_2_ dissolution, as discussed in the next section.

### Effective Transport Velocity

**Figure 9:**
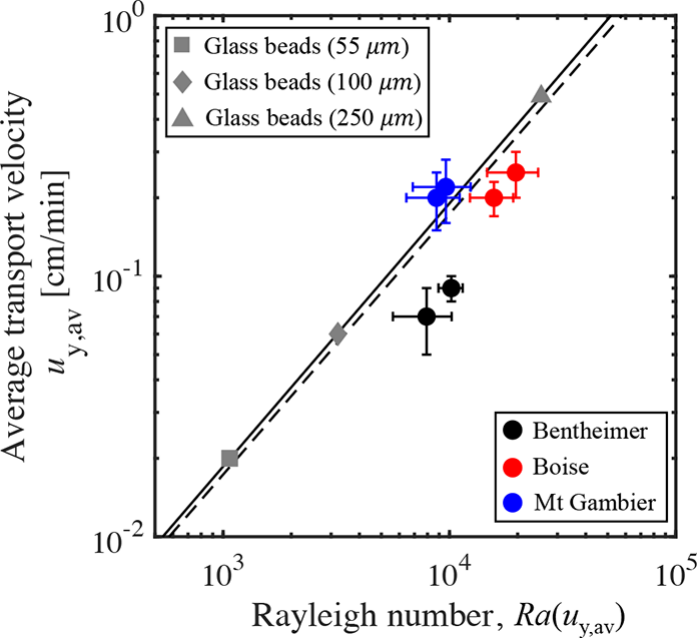
Mean transport velocity, *u*_*y*,av_, as a function of *Ra*(*u*_*y*,av_) for the free-solutal convection experiments with random bead packs (gray-filled symbols, three different bead sizes) and the three rocks (two repeats each). The solid line is a power law fit to the experiments with bead packs, i.e., *u*_*y*,av_ = *mRa*^*b*^, with *m* = 1.73 × 10^–5^ and *b* = 1.01 (*m* = 1.73 × 10^–5^ and *b* = 1 for the dashed line).

The complex relation between the texture of porous rocks and the resulting convective patterns challenges the definition of *Ra* that uses the buoyancy velocity *u*_B_ to normalize time for scaling purposes. While eq 2 incorporates the permeability of the given rock, it does not include any information on the morphology of the pore space, which affects the contribution of other transport mechanisms, such as dispersion or diffusion. Moreover, the downwelling plumes dilute with time, meaning that the “effective” transport velocity is substantially smaller than the buoyancy velocity *u*_B_, which uses a constant value of Δρ_max_ as the driver of the convective process. To address this aspect, we compute the transport velocity in the main direction of flow *u*_*y*_ from the displacement of the center of mass *Y*_com_(89):
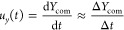
6whereby velocity *u*_*y*_ is computed during the advanced convective regime for each discrete time interval Δ*t* (solid lines in Figure 7). These values are then averaged to yield the mean transport velocity, *u*_*y*,av_. Accordingly, we define the Rayleigh number as
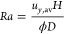
7The results of this analysis are reported in Table 3 and are depicted in Figure 9, where the mean transport velocity *u*_*y*,av_ is plotted as a function of *Ra*(*u*_*y*,av_). We observe that the three experiments with random cubic bead packs are closely aligned and follow a power-law behavior  with *b* = 1.01, i.e., reproducing the expected linearity (dashed line). The experiments with the three rocks do not follow the same trend line, confirming the qualitative observations from the X-ray images discussed above. Mt Gambier lies the closest to the trend line, while Bentheimer lies the farthest, again confirming the similarities (or lack thereof) in the evolution of the solute plumes between these rocks and the bead packs (see Figure 8). The lack of a universal scaling to describe the effective transport velocity during solutal convection in consolidated rocks challenges the use of laboratory-scale experimental data for up-scaling purposes.

**Table 3:** Rayleigh Number, *Ra*, and Average Transport Velocity *u*_*y*,av_ for the Experiments Presented in This Study Using Rocks and for a Previous Study Using Random Beadpacks with Distinct Bead Sizes^37^^,^^a^

		*Ra* (*u*_*y*,av_)	*u*_*y*,av_ [cm/min]
Bentheimer	#1	7924 (2 300)	0.09 (0.02)
	#2	10,188 (1 247)	0.07 (0.01)
Boise	#1	19,648 (4 987)	0.25 (0.05)
	#2	15,719 (3 405)	0.20 (0.03)
Mt gambier	#1	8765 (2 316)	0.20 (0.05)
	#2	9641 (2 756)	0.22 (0.06)
beads	55 μm	1069	0.02
	100 μm	3215	0.06
	250 μm	25,423	0.49

a The values in parentheses represent the computed uncertainties. For *u*_*y*,av_, the uncertainty is calculated as the standard deviation around the mean. The uncertainty in *Ra* is obtained upon the application of classic rules of error propagation.

In the discussion above, we have related the downward movement of the center of the solute mass to an effective constant transport velocity. In doing so, we have not considered the solute dispersion associated with this transport process. On the one hand, one could lump the effects of dispersion within an effective dispersivity coefficient.^37^ In fact, the complex porous structure of rocks gives reason to expect that spatial heterogeneities in the pore space intensify both the longitudinal and the transverse dispersive behavior. On the other hand, the interpretation (and scaling) of such coefficient does not appear straightforward, as in the experiments the volume-averaged mean fluid velocity is balanced around zero since the flow of the solute-rich downwelling plumes is opposed by the flow of the solute-lean upwelling plume. As such, these properties should be the focus of future investigations to further our understanding of free solutal convection and the associated solute dispersion process.

### Implications for CO_2_ Storage

The present study aims to support the selection of suitable sites for large-scale CO_2_ storage in saline aquifers. We found that highly porous rocks with strong (small-scale) heterogeneities show efficient mixing in all three spatial directions but convection slows down with distance traveled due to the reduction in the plume average solute density. On the other hand, more homogeneous rocks can sustain a strong downward convection, but show a less efficient spreading in the transverse direction. Therefore, we suggest that highly porous and heterogeneous rocks (such as Mt Gambier) are more favorable in saline aquifers with a shorter reservoir height because fast and efficient mixing within the large available pore space is expected. For aquifers with a deep confining bed, a homogeneous host rock of low- to medium-high porosity (such as the sandstones Bentheimer and Boise) could provide the advantage that plumes stay more compact, leading to greater amounts of CO_2_ transported into larger depths.

The results obtained in this study provide a major step toward a better understanding of the development of convective instabilities in different types of natural geological porous media. While laboratory studies are bound to under-represent geologic variability, their incorporation within digital rock workflows is disclosing new opportunities to improve predictions of fluid flow and transport across scales. In this context, the data generated in this work will be useful to inform 4D numerical studies of free solutal convection, which are just beginning.^75^ For subsurface CO_2_ storage, the gained knowledge on the complex behavior of the solute plume and its evolution will need to be implemented in, for instance, large-scale simulations of heterogeneous reservoirs that are used in the appraisal of the storage potential of candidate rock formations.

## Supporting Information Available

Additional experimental results as well as additional information on the experimental approach and analysis, including 3D X-ray CT tomograms of the rock samples; estimation of porosity and REV analysis; estimation of characteristic length scales, tortuosity, permeability; uncertainty analysis; and scaling of the spatial moments (PDF)
