# Supplementary material for: Direct Characterization of Free Solutal Convection in Porous Rocks for CO2 Storage Applications
Source: Environ Sci Technol. 2025 Mar 3;59(9):4618–30. doi: 10.1021/acs.est.4c10183 (PMC11912324; doi:10.1021/acs.est.4c10183)
Supplement: Supplementary file 1 — es4c10183_si_001.pdf [file es4c10183_si_001.pdf]

Supporting Information for ”Direct characterisation of free solutal convection in porous rocks for CO_2 storage applications“

Anna-Maria Eckel

Department of Chemical Engineering,

Imperial College London, SW7 2AZ London, United Kingdom

Current address: GFZ Helmholtz Centre for Geosciences,

Section 4.3, Geoenergy, Telegrafenberg, 14473 Potsdam, Germany

Andrea Rovelli

Department of Chemical Engineering,

Imperial College London, SW7 2AZ London, United Kingdom

Ronny Pini

Department of Chemical Engineering,

Imperial College London, SW7 2AZ London, United Kingdom

Corresponding author: r.pini@imperial.ac.uk

This document includes: 19 pages, 9 figures, 1 table.

INTRODUCTION

The supporting information includes text, figures and tables which supports the main publication in explanations or conclusions (referred to as SX in the main publication). The data was created along with the data shown in the main publication.

Figure S1: 3D X-ray CT tomograms of the rock samples

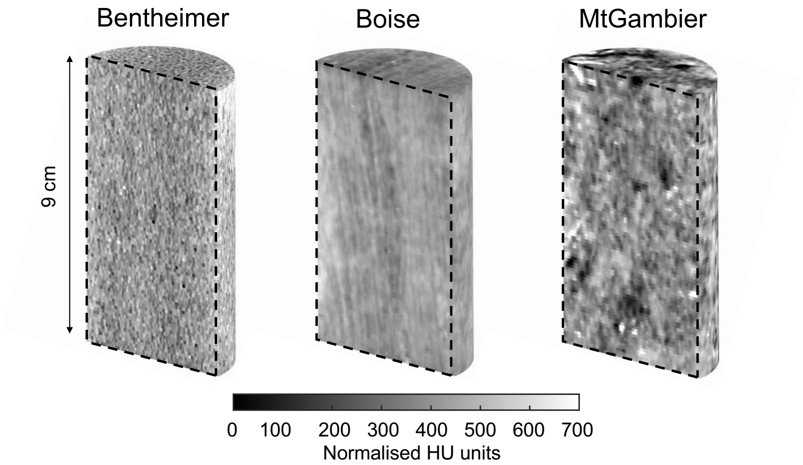

FIG. S1: 3D X-ray CT tomograms of the three rock samples used in this study. The grey scale represent normalised Hounsfield units, which can be used as proxy for the bulk density. Images are oriented as in the free solutal convection experiments.

Text S1: Estimation of Total Porosity and its Uncertainty

To obtain the total porosity ϕ_t of the rocks (core samples of dimensions 15 cm length and 2-inch diameter), we applied the X-ray saturation technique [1]. Two end-caps are secured on each face of the sample and secured with a shrink tube (51 mm sleeve diameter, purchased from TE Connectivity) by carefully applying heat all around. After placing them on the medical CT scanner bed, the rocks were first scanned in air-saturated condition. The images were reconstructed at a spatial resolution of 0.5 mm in the z-direction and roughly 0.1–0.2 mm in the x- and y-direction (in-plane resolution). This value changes slightly between the different samples used. The sample is then flushed with gaseous CO_2 for several minutes and afterwards saturated with water (7–8 pore volumes). The samples are again scanned in the same position, this time in water-saturated condition. The following equation is applied for the calculation of the total (connected) porosity on voxel-basis [1, 2]:

$$\phi_{t,i} = \frac{CT_{wet,i} - CT_{dry,i}}{CT_w - CT_{air}}. \tag{1}$$

$CT_w = 40\,\text{HU}$ and $CT_{air} = -976\,\text{HU}$ are the CT numbers (in Hounsfield units) of pure water and air. $CT_{dry,i}$ and $CT_{wet,i}$ are the CT numbers of a voxel when i the rock is saturated with either air or water. The obtained CT-images have been resampled with a coarsening scheme of 30×30 (initial voxel dimensions: $(0.1\times0.1\times0.5)\,\text{mm}^3$ to $(3\times3\times0.5)\,\text{mm}^3$) which reduces the uncertainty on the voxel scale significantly [1]. The total porosity is the sample-average of the voxel porosities ($\phi_t = \sum \phi_{t,i}/N$, with N being the number of voxels) and the values are listed in Table 1 of the main manuscript.

The porosity values predicted using Eq. 1 are affected by an uncertainty ($\sigma_{\phi_{t,n=2}}$). This uncertainty is calculated from the "CT noise" $\sigma_{\Delta CT_{n=2}}$ by subtracting $n = 2$ coarsened images of the dry rock. We calculate the uncertainty with [3]:

$$\sigma_{\phi_{t,n=2}} \approx \frac{\sigma_{\Delta CT_{n=2}}}{1000}. \tag{2}$$

and the values, given in brackets in Table 1 of the main manuscript, are all around $\sigma_{\phi_{\mathrm{t,n=2}}} = 1\,\%$. The value in the denominator results from the combination of dry and wet images, *i.e.*, the difference of the CT numbers of the two pure reference fluids, water ($CT_{\mathrm{w}} - CT_{\mathrm{air}} \approx 1000\,\mathrm{HU}$). More details on the derivation of Eq. 2 can be found in [3].

Text S2: Estimation of Mobile Porosity and REV Analysis

Although the micro CT resolution is very high, pores smaller than the voxel size ($< 3.9\,\mu\mathrm{m}$) or other small features cannot be resolved. Thus, we interpret the porosity values extracted from the micro CT as the mobile porosity ϕ. First, the micro CT images are binarised using an interactive thresholding operation in the image analysis software AVIZO-9 (ThermoFisher Scientific). The threshold for each rock type is chosen based on the minimum between the high- and the low-intensity peak in the image histogram and validated by qualitatively comparing cross-sectional greyscale images with the computed binary images. The determined threshold for each rock is given in Table S1.

TABLE S1: Threshold of the binarisation of the micro CT images applied in AVIZO-9. The values are valid for the files in tiff-format while 0 represents the minimum intensity, and 65 535 represents the maximum intensity.

Rock sample	threshold
Bentheimer	12 900
Boise	13 100
Mt Gambier	16 670

Exemplary cross-sections of the cube samples are shown in Figure S2. The mobile porosity is calculated by taking the ratio of the number of voxels labelled to be part of

the pore space N_{pore} divided by the total number of voxels N_{total} (see Table 1 in main manuscript):

$$\phi = \frac{N_{\text{pore}}}{N_{\text{total}}}. \tag{3}$$

The estimation of the uncertainty of the mobile porosity is given in Text S7.

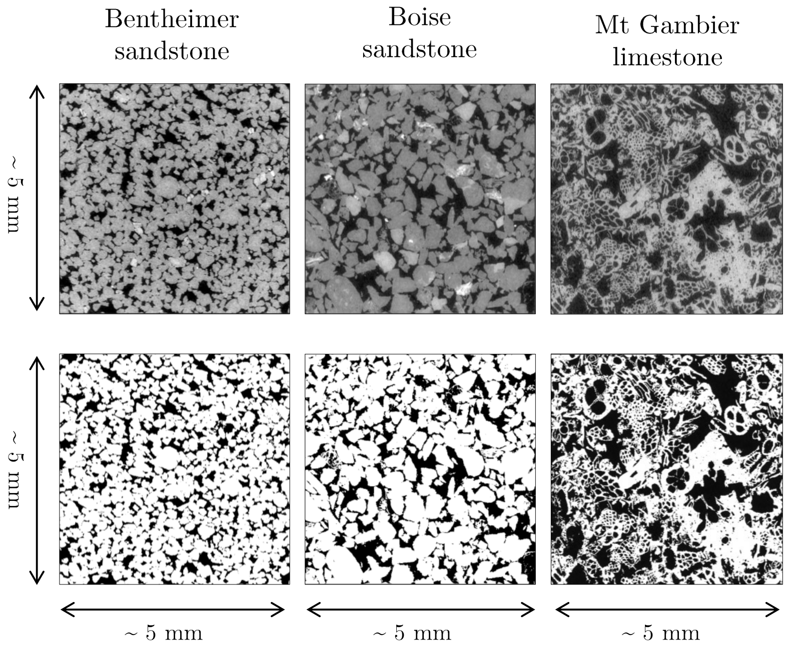

FIG. S2: Top images: cross-sections taken from the micro CT images, bottom images: binarised version of the same sections (back is pore space, white is rock material).

To determine a representative sample size for characterizing the properties of the rocks, we performed a Representative Elementary Volume (REV) analysis on binary images obtained from scanning a rock sample plug in the micro-CT scanner (sub-sample B in Figure S3). The REV analysis was conducted by using the mobile porosity as a parameter of interest because it is an additive property and therefore suitable for upscaling by a simple averaging scheme. A classical REV estimation has been applied using point-centred cube subsamples. The edge length of the subsample volume is calculated to $L = 3\sqrt[3]{V}$ [4]. The mobile porosity ϕ is shown as a function for the cube length L in Figure S4a. The REV ($= V_{\mathrm{min}}$) is determined using the criterion for the porosity after [5] reported in [4]. It uses the relative gradient error ε_{g} and is shown plotted in Figure S4b. More details are given in the figure caption.

The REV ranged between 1.5 and 1.7 mm for all three rock types. Based on these findings, we used an image volume of $5\times5\times5\,\mathrm{mm}^3$ (sub-sample C) to determine the characteristic properties (mobile porosity, tortuosity, and characteristic length) for each sample. For each rock type, at least two sub-samples C from different locations within sub-sample B were used to estimating these properties.

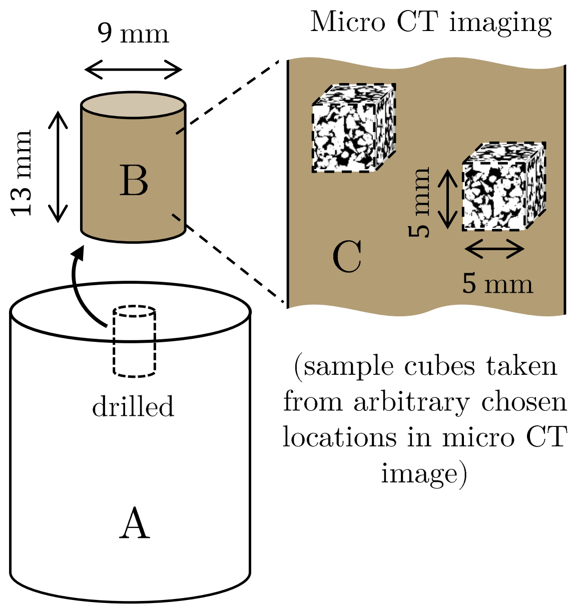

FIG. S3: Schematic representation of sample sizes. Samples B were drilled from a larger core (A) using a Scheppach Top Pillar DP16SL bench drill (1 cm diameter drill bit). Cubes C (two for each rock type, three for Mt Gambier) were cropped out of the micro CT images taken of sample B at arbitrarily chosen locations.

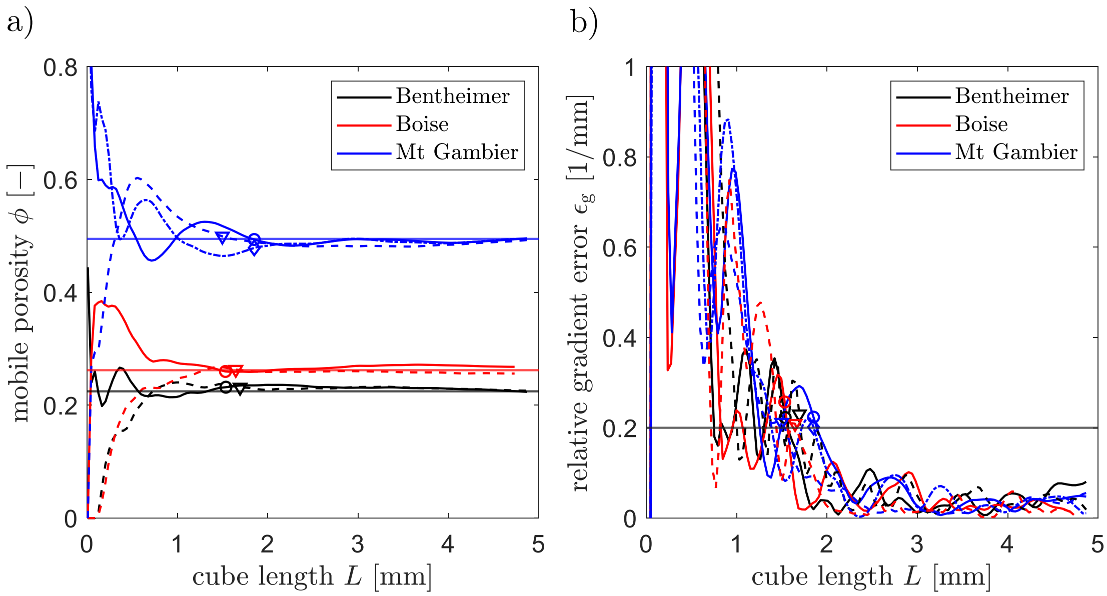

FIG. S4: Variability (a) and relative error gradient (b) of the average mobile porosity of the point-centred cube sample with side length L for 125 realisations. The data is colour-coded to represent different rock types (Bentheimer in black, Boise in red, Mt Gambier in blue). The colour-coded solid and dashed lines represent the results using the different samples C (one additional for Mt Gambier as a dash-dotted line). The symbols mark the determined REV where ε_g falls below 0.2 according to the criterion (grey solid line in b).

Text S3: Estimation of Charactersitic Length

A common approach to estimate the characteristic length of a porous medium is to use the diameter of rock grains or synthetic beads ($d_{p,50}$). However, in some cases, it is difficult to unambiguously identify individual grains. Additionally, the fossiliferous Mt Gambier limestone does not show a grain structure. Hence, we follow the approach described in [6, 7] to calculate a physical representative grain size L_c based on a cubic packing of regular spheres. They calculate the characteristic length with $L_c = \pi V/S$ with V as the volume of the porous medium (solid and pore space) and S as the area of the pore-solid interface. For all rocks, the average of the characteristic length and the uncertainty were calculated using two samples, solely for Mt Gambier three samples were used to reduce the uncertainty (uncertainty is the standard deviation of all values computed).

An image processing routine was developed in the MATLAB environment and the relevant steps are illustrated in Figure S5 by means of one sandstone (Bentheimer) and the carbonate (Mt Gambier). Details on the routine are given in the figure caption.

After importing the binary images of the samples into the software (images on the left), in the first step, the unconnected pixels are cleaned by a morphological operation (images in the middle). The perimeter of the rock solid-phase is determined (images on the right panel) and the sum of the perimeter voxels is assumed to be the pore-solid interface ($S = \sum N_{\mathrm{peri}}\, l_{\mathrm{vox}}$, with N_{peri} as the number of perimeter voxels and l_{vox} as the voxel side length). The estimation of the uncertainty of the characteristic length scale is given in Text S7. The values of the characteristic length L_c are shown in Table 1 of the main manuscript together with uncertainty values.

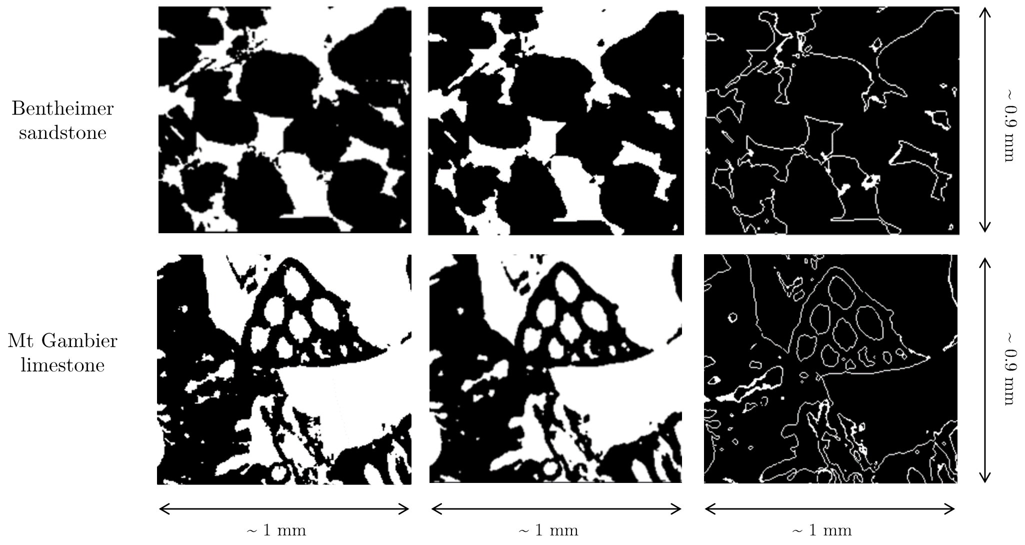

FIG. S5: Steps of the MATLAB routine to extract the pore-solid interface for the calculation of the characteristic length. The routine is shown exemplarily for one sandstone (Bentheimer) and one carbonate (Mt Gambier). The two images on the left show a section of the binarised micro CT image (solid black, air white), the images in the middle are cleared with morphological operations that only retain voxels if more than half of the voxels in the neighbourhood of the target voxel are set to 1. The images to the right show the detected perimeter (white) around the solid phase (back). A voxel is part of the perimeter if it is non-zero and connected to at least one zero-valued pixel and voxels are connected if their faces touch.

Text S4: Estimation of Tortuosity

We are applying *TauFactor* [8, 9], an open-source MATLAB application, that used finite difference-type discretisation to solve Fick's law of diffusion to approximate the tortuosity factor of the rock samples. The tortuosity factor reported in Table 1 of the main manuscript is the average of 6 values (2 representative cubic 3D micro CT images, 3 directions each) and 9 values for Mt Gambier (3 representative cubes, 3 directions each). The estimation of the uncertainty of the mobile porosity is given in Text S7.

Text S5: Estimation of Permeability and its Uncertainty

The absolute permeability k of each rock sample is determined during steady-state brine injection by measuring the flow rate Q and the mean pressure drop Δp at various flow rates. The permeability can be extracted according to Darcy's law [10]:

$$\frac{Q\mu L}{A} = k\Delta p. \tag{4}$$

The measurements were performed at ambient pressure and temperature conditions. The experimental apparatus consists of a custom-built aluminium core holder containing the rock samples. The sample was wrapped in a shrink tube to prevent fluid loss. A constant confining annular pressure between the jacketed sample was maintained with a high-pressure syringe pump (Teledyne ISCO, Model 1000D) and tap water as the confining fluid. A differential pressure transducer (Keller UK, model PRD-33X) is connected to the inlet and outlet faces of the sample. A back pressure of roughly 8 bar was employed on the outer face of the sample. The system was purged with gaseous CO_2 and then saturated with brine (3.7 % NaCl brine). Various flow rates were applied and the associated pressure drop along the core was measured. The pressure drop is corrected with a correction factor which is equal to the measured pressure drop when the flow is turned off. The measured

pressure drop Δp^* is assumed to be equal to the true pressure drop Δp plus the correction factor Δp_f:

$$\Delta p^* = \Delta p + \Delta p_\mathrm{f}. \tag{5}$$

In Figure S6, the flow rate as $Q\mu L/A$ is plotted against the corrected (true) pressure drop Δp.

The absolute values of k and its uncertainty are determined using weighted linear regression of the flowrate as a function of the pressure drop across the core samples

Figure S6: Estimation of Permeability

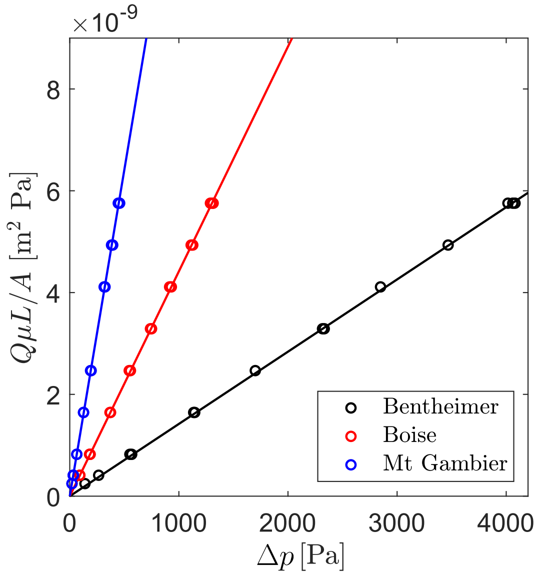

FIG. S6: Flow rate $Q\mu L/A$ versus pressure drops Δp across the core samples during multi-rate water injection tests. Once the pressure drop is stabilised, pressure values are reported over an average of at least 5 minutes. The symbols correspond to the experimental data and the solid lines are the weighted linear regression to the experimental measurements. Darcy's equation (Eq. 4) is used to estimate the permeability k of the samples. The viscosity of the brine takes a value of 1×10^{-3} Pas. The absolute permeability values are given in Table 1 of the main manuscript.

Text S6: Uncertainty of CT values, KI Mass Fraction and KI Molar Concentration

We follow the procedures described in detail in [11] to estimate the uncertainty of the CT values caused by quantum mottle and to calculate the uncertainty of the molar concentration. The uncertainty of the CT number caused by quantum mottle was estimated using scans of the saturated rocks taken prior to the start of the experiment and calculated to $\sigma_{\Delta_{\mathrm{CT}}} = 18\,\mathrm{HU}$ (Bentheimer), $\sigma_{\Delta_{\mathrm{CT}}} = 19\,\mathrm{HU}$ (Boise) and $\sigma_{\Delta_{\mathrm{CT}}} = 16\,\mathrm{HU}$ (Mt Gambier). Using statistical methods of error propagation, the uncertainty of the computed mass fraction of solute is estimated to be between $\sigma_{\mathrm{w_i}=0} = 0.01$ (pure water) and $\sigma_{\mathrm{w_i}=0.6} = 0.09$ (maximum saturation). Accordingly, the uncertainty in the computed concentration of solute is estimated to be $\sigma_{\mathrm{c_i}} = 46$ mol/m^3 (pure water) and $\sigma_{\mathrm{c_i}} = 1\,421$ mol/m^3 (saturated solution, $c_{\mathrm{max}} = 6\,198$ mol/m^3). The presented uncertainty values are calculated for Bentheimer but the values of the other rocks are very similar or smaller.

Text S7: Uncertainty of Mobile Porosity, Tortuosity and Characteristic Length-Scale

Uncertainties on the estimates of mobile porosity, ϕ, the pore-space tortuosity, τ, and of the characteristic length-scale represent absolute differences between measurements on different sister samples. The binarised microCT images are also used to obtain estimates of the pore-space tortuosity, τ, and its uncertainty by using the open-source code *TauFactor* [8, 9]. The values of τ and its uncertainty (standard deviation) are computed out of 6 (or 9 for Mt Gambier) estimated values for the tortuosity, thereby covering 3 spatial directions.

Text S8: Estimation of Diffusion Coefficient

The diffusion coefficient of water with KI content is computed with data from [12]. The data is measured at 296.15 K and KI concentrations from 0 mol/m^3 and 3400 mol/m^3.

An average of those values is taken which results in $D_\mathrm{m} = 2.61 \times 10^{-9}\,\mathrm{m}^2\ \mathrm{s}^{-1}$ and used as a molecular diffusion coefficient in the main manuscript.

Figure S7: Time Normalisation

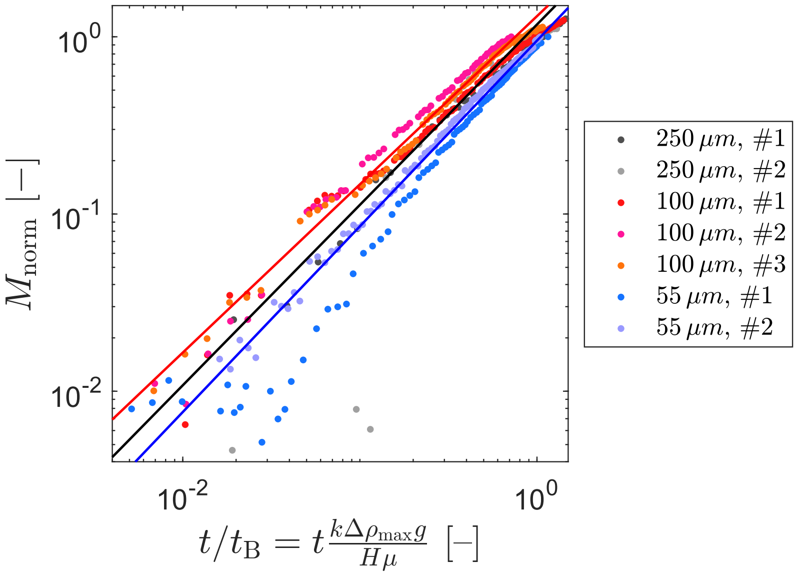

FIG. S7: Normalised total mass M_norm of the bead packs experiments plotted as a function of the normalised time $\tilde{t} = t/t_\mathrm{B}$. The solid lines are obtained upon fitting a power law function to the experimental data in the convective regime. The curves are very close together but show a slight horizontal shift between the trendlines. This could be a result of a small discrepancy between the calculated and the real permeability values of the bead packs, *e.g.* arising from packing inconsistencies.

Figure S8: Spatial Moments of Solute Mass

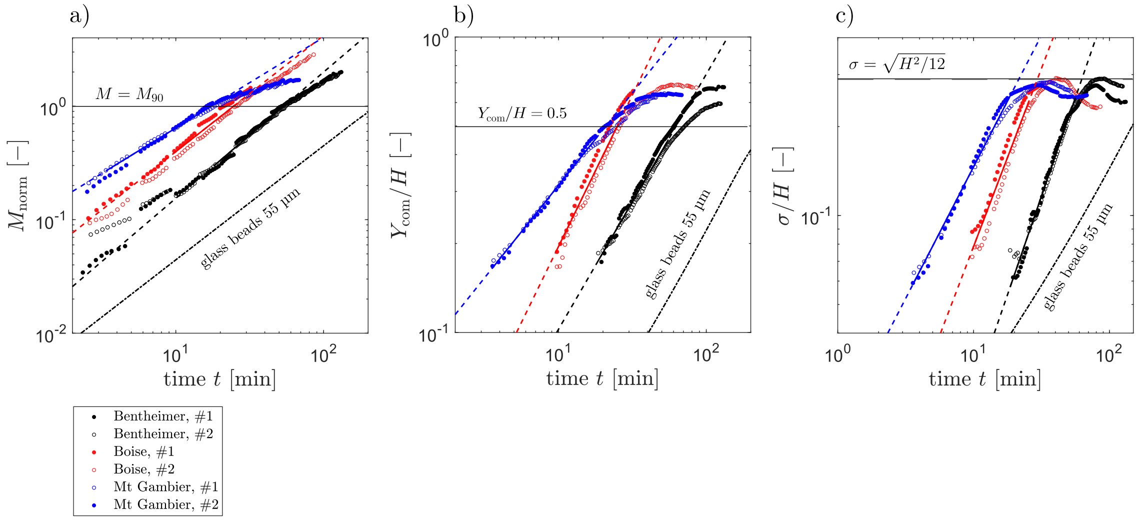

FIG. S8: Spatial moments of the solute mass plotted as function of absolute time t for the three rock samples. (a) Zeroth moment represented as normalised total mass, $M_{\mathrm{norm}} = (M(t) - M_0)/(M_{90} - M_0)$. M_0 and M_{90} refer to the start of the experiment and the attainment of near-complete mixing (i.e. $\sigma = 0.9\sigma_{\mathrm{max}}$, where $\sigma_{\mathrm{max}} = \sqrt{H^2/12}$ and $Y_{\mathrm{norm}} = H/2$), respectively. (b) First moment represented as the dimensionless vertical centre of mass Y_{norm}/H, where H the sample height. (c) Second moment represented as the dimensionless standard deviation of the solute concentration distribution around the mean in the vertical direction, σ/H. Empty and filled symbols refer to experiments conducted on different samples of the same rock type. The solid lines (colour-coded) are power-law fits to the experimental data in the convective regime (the line is dashed outside this regime). The dash-dotted line refers to results of experiments conducted using random beadpacks (bead size 55 μm, $Ra = 3000$, from [11]).

Figure S9: Scaling of the zeroth spatial moment

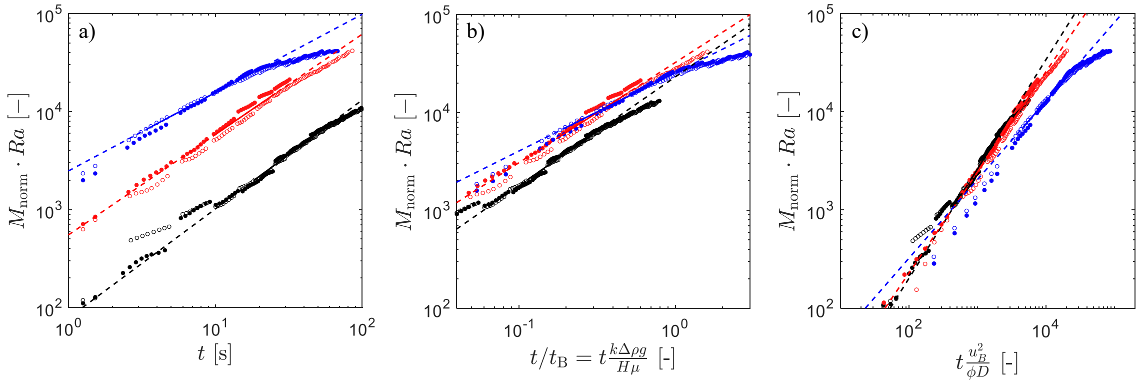

FIG. S9: Three different representations of the temporal evolution of the zeroth spatial moment of the solute mass. Differently from Figure 7a of the main manuscript, $M_{\rm norm}$ is rescaled with respect to the Rayleigh number, Ra, i.e. $M_{\rm norm} \cdot Ra$ (panel a). Panel b and c use a normalised time, $t/t_{\rm B}$ and two different characteristic length scales L to define the characteristic time scale, $t_{\rm B} = L/u_{\rm B}$, namely $L = H$ (panel b) and $L = \phi D/u_{\rm B}$ (panel c).

REFERENCES

[1] Ronny Pini and Claudio Madonna. Moving across scales: a quantitative assessment of X-ray CT to measure the porosity of rocks. *Journal of Porous Materials*, 23(2):325–338, 2016.

[2] Takeshi Kurotori, Christopher Zahasky, Sayed Alireza Hosseinzadeh Hejazi, Saurabh M. Shah, Sally M. Benson, and Ronny Pini. Measuring, imaging and modelling solute transport in a microporous limestone. *Chemical Engineering Science*, 196:366–383, 2019.

[3] Ronny Pini, Samuel C.M. Krevor, and Sally M. Benson. Capillary pressure and heterogeneity for the CO_2/water system in sandstone rocks at reservoir conditions. *Advances in Water Resources*, 38:48–59, 2012.

[4] Molly S. Costanza-Robinson, Benjamin D. Estabrook, and David F. Fouhey. Representative elementary volume estimation for porosity, moisture saturation, and air-water interfacial areas in unsaturated porous media: Data quality implications. *Water Resources Research*, 47(7), 2011.

[5] J. H. Li, L. M. Zhang, Y. Wang, and D. G. Fredlund. Permeability tensor and representative elementary volume of saturated cracked soil. *Canadian Geotechnical Journal*, 46(8):928–942, 2009.

[6] Branko Bijeljic, Peyman Mostaghimi, and Martin J. Blunt. Insights into non-Fickian solute transport in carbonates. *Water Resources Research*, 49(5):2714–2728, 2013.

[7] Branko Bijeljic, Peyman Mostaghimi, and Martin J. Blunt. Signature of non-Fickian solute transport in complex heterogeneous porous media. *Physical review letters*, 107(20):204502, 2011.

[8] Samuel Cooper. Taufactor: App for analysis of image based geometry data in terms of tortuosity factors, volume fractions, surface areas and triple phase boundaries. Samuel Cooper (2023). TauFactor (https://www.mathworks.com/matlabcentral/fileexchange/57956-taufactor), MATLAB Central File Exchange, 2016.

[9] S. J. Cooper, A. Bertei, P. R. Shearing, J. A. Kilner, and N. P. Brandon. Taufactor: An open-source application for calculating tortuosity factors from tomographic data. *SoftwareX*, 5:203–210, 2016.

[10] Colin McPhee, Izaskun Zubizarreta, and Jules Reed, editors. *Core analysis: A best practice guide*, volume volume 64 of *Developments in petroleum science*. Elsevier, Amsterdam, Netherlands, 2015.

[11] Anna-Maria Eckel, Rebecca Liyanage, Takeshi Kurotori, and Ronny Pini. Spatial moment analysis of convective mixing in three-dimensional porous media using X-ray CT images. *Industrial & Engineering Chemistry Research*, 62(1):762–774, 2023.

[12] David W. McCall and Dean C. Douglass. The effect of ions on the self-diffusion of water. I. Concentration dependence. *The Journal of Physical Chemistry*, 69(6):2001–2011, 1965.
